# Genome-wide association study for the extractable phenolic profile and coat color of common bean seeds (*Phaseolus vulgaris* L.)

**DOI:** 10.1186/s12870-023-04177-z

**Published:** 2023-03-23

**Authors:** Ana Campa, Roberto Rodríguez Madrera, María Jurado, Carmen García-Fernández, Belén Suárez Valles, Juan José Ferreira

**Affiliations:** Regional Service for Agrofood Research and Development (SERIDA), Ctra AS-267 PK 19, 33300 Villaviciosa, Asturias Spain

**Keywords:** *Phaseolus vulgaris*, Common bean, Genome-wide association study, Phenylpropanoid pathway, Seed coat color genes, Candidate genes

## Abstract

**Background:**

A large variation in seed coat colors and seed phenolic metabolites is present in common bean (*Phaseolus vulgaris* L.). The study of the relationships between seed coat color phenotype and the phenolic profile is an important step in the elucidation of the gene network involved in the phenylpropanoid biosynthetic pathway. However, this relationship is still poorly understood in this species.

**Results:**

A genome-wide association study (GWAS) was used to investigate the genomic regions associated with the synthesis of 10 flavonoids (5 anthocyanins and 5 flavonols) and with 10 seed coat color traits using a set of 308 common bean lines of the Spanish Diversity Panel (SDP) which have been genotyped with 11,763 SNP markers.. A total of 31 significant SNP-trait associations (QTNs) were identified, grouped in 20 chromosome regions: 6 for phenolic metabolites on chromosomes Pv01, Pv02, Pv04, Pv08, and Pv09, 13 for seed coat color on chromosomes Pv01, Pv02, Pv06, Pv07, and Pv10, and 1 including both types of traits located on chromosome Pv08. In all, 58 candidate genes underlying these regions have been proposed, 31 of them previously described in the phenylpropanoid pathway in common bean, and 27 of them newly proposed in this work based on the association study and their homology with *Arabidopsis* anthocyanin genes.

**Conclusions:**

Chromosome Pv08 was identified as the main chromosome involved in the phenylpropanoid pathway and in consequence in the common bean seed pigmentation, with three independent chromosome regions identified, Phe/C_Pv08(2.7) (expanding from 2.71 to 4.04 Mbp), C_Pv08(5.8) (5.89–6.59 Mbp), and Phe_Pv08(62.5) (62.58 to 63.28 Mbp). Candidate genes previously proposed by other authors for the color genes *V* and *P* were validated in this GWAS. Candidate genes have been tentatively proposed from this study for color genes *B* and *Rk* on Pv02, *Asp* on Pv07, and complex *C* on Pv08. These results help to clarify the complex network of genes involved in the genetic control of phenolic compounds and seed color in common bean and provide the opportunity for future validation studies.

**Supplementary Information:**

The online version contains supplementary material available at 10.1186/s12870-023-04177-z.

## Background

Common bean (*Phaseolus vulgaris* L.) is among the most important food legumes for human consumption globally. Nutritionally, common bean seeds represent an important protein source in sustainable agri-food systems [1, 2]. Depending on their genotype, common bean seeds can also carry a variety of phenolic compounds, produced as secondary metabolites, that show important human health benefits by acting as antioxidants, anticarcinogens, and anti-inflammatories [3, 4]. Also, important agronomic benefits have been related to the phenolic profile, such as the link between genotypes with high phenolic concentrations and greater resistance to plant diseases [5].

Among phenolic compounds, flavonoids (flavonol glycosides, anthocyanins, and condensed tannins) are derived from phenylpropanoid metabolism and are mainly located in the seed coat. Flavonoid concentration and profile vary widely depending on the genotype and are responsible for the seed coat color. Dark seeds, like black or red, normally have the highest anthocyanins content, proanthocyanidins are responsible for brown pigments and pale-yellow or cream is due to the presence of flavonol glycosides [6–9]. Pure white-seeded genotypes lack flavonols, while seeds with colored patterns have varying amounts of flavonols depending on the proportion and type of color [9]. Most of the current knowledge about phenylpropanoid synthesis and its genetic control in common bean has been inferred from other plant systems such as *Arabidopsis* [10] or soybean [11, 12]. The study of the relationships between seed coat color genes and flavonoid content is an important step in the elucidation of genes involved in the phenylpropanoid biosynthetic pathway, but this relationship is still poorly understood in common bean. Understanding the function of these genes could have implications for human health, but also for the common bean response to biotic stresses, since genotypes showing dark seed colors have been related with higher resistance to diseases [13, 14].

The inheritance of seed coat color in common bean has been studied since the early twentieth century [15–18] because consumers associate seed color and patterns with differences in processibility, flavor, and texture, but also due to its importance in defining market classes [19]. A complex network of epistatic genes has been described in the expression of the seed coat color through classic genetic analysis, including color-determining genes, modifiers, and/or color pattern genes. Most of these genes have been located in the common bean genome. The *P* gene (chromosome Pv07) is a primary basic gene considered as the control factor for the presence or absence of color, whose dominant genotypes show a colored seed coat while recessive genotypes prevent color expression and produce a white coat [20, 21]. Other color genes described are *J* (= *L* gene, mature color development on Pv10), *G* (yellow–brown, Pv04), *B* (gray-brown, Pv02), *V* (violet factor, Pv06), complex *C* locus (complete color; Pv08), *Rk* (red kidney) whose location is controversially being located on both Pv01 [22], and Pv02 [11, 17], *Z* (= *D*, zonal pattern color, Pv03), *T* (totally pattern color, Pv09), *Gy* (Grenish-yellow pattern, Pv08), and *Bip* (coat patterns based on the hilum, Pv10) [11, 23].

Several associations have been described in common bean between the seed coat color and the flavonoid genes with varying degrees of accuracy. The *B* gene, responsible for gray-brown colors on Pv02, was proposed to regulate the production of anthocyanin precursors in the seed coat color pathway above the level of dihydrokaempferol formation at the chalcone synthase or chalcone isomerase step [24]. Although the *Asp* gene is not involved in the color, it is responsible for the seed coat glossiness or shine and it was identified as a gene involved in the phenylpropanoid pathway by limiting the amounts of anthocyanins present in the coat [24]. Caldas and Blair [25] using three RIL populations identified 12 quantitative trait loci (QTL) for condensed tannin, some of them located in regions containing the color genes *Z* on Pv03, *V* on Pv06, *P* on Pv07, *C* on Pv08, and *Bip* on Pv10. Candidate genes have been proposed only in some specific cases. Reinprecht et al. [11] based on comparative genomic analysis with soybean (*Glycine max* L. Merr) located 46 phenylpropanoid pathway genes and identified potential associations with seed coat color genes: a gene coding the enzyme 4-coumarate: CoA ligase mapped close to the *Z* color gene on chromosome Pv03, and the *Myb15* transcription factor mapped close to the *P* gene on Pv07. Later, the *P* gene was confirmed to be a basic helix-loop-helix (*bHLH*) *MYB* transcription factor, with two candidate genes, *Phvul.007G171333* and *Phvul.007G*171466, that allows or blocks the expression of other color genes [21]. García-Fernandez et al. [26] using a RIL population derived from the cross TUxMUSICA identified two candidate genes controlling black seed coat color, *Phvul.006G018800* encoding an *F3´5´H* on Pv06 that correspond to *V* color gene [27], and *Phvul.008G038400* encoding a *MyB113* transcription factor on Pv08 that could correspond to the *C* color locus.

Genome-wide association study (GWAS) is a powerful tool for identifying and mapping genes or QTL involved in the genetic control of important traits. GWAS of phenolics has been conducted in non-legume crops like cereals [28], rice [29], and tomato [30]. In common bean, GWAS have been conducted for total phenolic content in the pod [31], but not in the seed. The Spanish Diversity Panel (SDP) is formed by 308 lines considered representative of the Spanish diversity for this species [32] and which were recently characterized for their seed extractable phenolic profiles and antioxidant activities [9, 33]. The objective of this work was to conduct a GWAS using the SDP to identify genomic regions associated with seed extractable phenolic compounds and determine whether color genes are located or related to these genomic regions. Adding knowledge to the relationship between phenolic compounds and seed colors has important implications for plant breeding, for the improvement of product nutritional qualities, and plant disease resistance.

## Results

### Seed coat phenolic metabolites

Table 1 shows the observed variation for 13 anthocyanins and 15 flavonols (see Additional file 1). The most frequently detected anthocyanin was Cya_G (36.5%), while Cya_diG was the anthocyanin detected with less frequency (1.5%). Concerning quantity, Del_G anthocyanin was detected in the highest level, ranging from 0 to 871.25 µg/g, while Cya_GMal was detected in low concentrations, ranging from 0 to 14.84 µg/g. The most frequent flavonol was Que_G (64.6%), while the less frequent flavonol was Myr_XylG (2.9%). With respect to quantities, Kae_G showed the highest concentration, from 0 to 2042.41 µg/g, while Kae_Rut was present in low concentrations, ranging from 0 to 3.84 µg/g.

**Table 1:** Observed variation for 13 anthocyanins and 15 flavonols in the Spanish Diversity Panel. Phenolic compound values are indicated in µg/g. Mean, standard deviation (SD), minimum (Min), and maximum (Max) values are indicated. Freq, frequency of the phenolic compound in the Spanish Diversity Panel

Phenolic compound	Abbreviation	Freq (%)	Mean	SD	Min–Max
*Anthocyanins*					
cyanindin3-O-glucoside	Cya_G^a^	36.5	11.69	35.48	0—334.29
cyanindin3-O-diglucoside	Cya_diG	1.5	0.32	2.78	0—28.72
cyanindin3-O-6-malonylglucoside	Cya_GMal	3.3	0.31	1.74	0—14.84
delphinidin3-O-glucoside	Del_G^a^	19.0	42.24	127.05	0—871.25
delphinidin3,5-O-diglucoside	Del_diG	9.1	1.28	4.38	0—30.15
malvidin3-O-glucoside	Mal_G^a^	5.8	2.48	12.34	0—93.42
malvidin3,5-O-diglucoside	Mal_diG	5.5	1.31	6.15	0—45.08
pelargonidin3-O-glucoside	Pel_G^a^	28.5	10.54	27.49	0—227.31
pelargonidin3,5-O-diglucoside	Pel_diG	4.4	0.48	2.32	0—15.28
pelargonidin-O-pentosidehexoside	Pel_PenHex	2.9	0.31	1.85	0—15.41
pelargonidin3-O-6malonylglucoside	Pel_GMal	12.4	1.20	3.40	0—26.32
petunidin3-O-glucoside	Pet_G^a^	9.9	7.85	32.86	0—258.33
petunidin3,5-O-diglucoside	Pet_diG	5.1	1.59	7.69	0—51.13
*Flavonols*					
kaempferol	Kae	45.6	4.48	14.57	0—158.46
kaempferol3-O-glucoside	Kae_G^a^	62.4	178.37	376.12	0—2042.41
kaempferol3-O-xyloglucoside	Kae_XylG	36.1	9.97	32.36	0—300.66
kaempferol3-rutininoside	Kae_Rut	11.3	0.10	0.41	0—3.84
kaempherol3-O-acetilglucoside_I	Kae_GAcI	34.7	1.98	3.81	0—19.20
kaempherol3-O-acetilglucoside_II	Kae_GAcII^a^	53.6	46.64	82.79	0—454.69
myricetin	Myr	11.7	0.32	1.79	0—21.47
myricetin3-O-glucoside	Myr_G^a^	24.1	13.00	45.61	0—300.29
miricetin3-O-xyloglucoside	Myr_XylG	2.9	0.09	1.02	0—16.57
myricetin3-O-malonylglucoside	Myr_GMal	10.6	0.41	1.65	0—15.09
quercetin	Que	31.0	0.79	2.64	0—29.79
quercetin3-Oglucoside	Que_G^a^	64.6	16.56	30.73	0—165.21
quercetin3-Oxyloglucoside	Que_XylG	16.1	0.52	2.05	0—18.00
quercetin3-O-rutininoside	Que_Rut	53.6	0.72	1.69	0—18.80
quercetin3-Oacetilglucoside	Que_GAc^a^	58.8	4.83	11.54	0—113.60

^a^Phenolic compounds considered for the GWAS

Eleven lines of the SDP were randomly selected and grown in 2019 to reanalyze their phenolic profiles for comparison to the characterization conducted in 2018. Lines included in this subsample were heterogeneous, showing significant differences between them for the phenolic profile (ANOVA; *F* = 2.614, *p* < 0.01), but significant differences between annuities were not observed (ANOVA; *F* = 0.096, *p* > 0.05). This result suggests the stability of the phenolic characterization between growing seasons.

### Seed coat color

Seed coat color was characterized based on the CIELAB vectors L*, a*, and b* (Additional file 1). L* ranges from 27.63 to 86.17, a* ranged from -4.63 to 18.06, and b* ranged from 1.41 to 36.33. Seed coat color was also characterized based on a qualitative classification into 6 main colors. The most frequent color was white (93 accessions), followed by cream (58), black (42), red (40), brown (37), and yellow (20). A total of 65 accessions showed color patterns including partly colored, mottled, or color in the hilum ring.

### Phenolic profile and color relationship

**Fig. 1:**
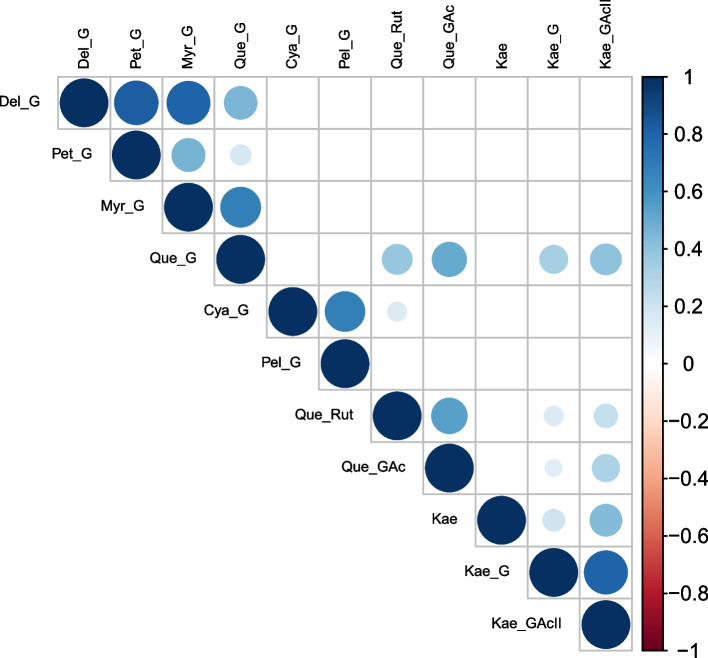
Spearman´s correlation coefficient between the seed coat color vectors L*, a*, b*, and the 10 phenolic profiles considered. Empty squares: not significantly associated

Figure 1 shows the Spearman correlations between the quantitative traits L*, a*, b* and the metabolites. The color vector L* is significantly and negatively associated with all traits considered except with b*, with whom it is significantly and positively associated. The color vector b* is significantly associated with all traits except with Que_G and Que_GAc. The color vector a* is significantly associated with all traits except Myr_G. Three main groups of metabolites were significantly and positively correlated: the 4 metabolites derived from the aglycones quercetin, (Que_G, Que_GAc) and kaempferol (Kae_G, Kae_GAcII); the metabolites Mal_G, Myr_G, Del_G, and Pet_G; and the anthocyanins Cya_G and Pel_G.

ANOVA and Tukey tests were conducted between color groups and phenolic metabolite content to detect significant differences between seed color classes (Table 2). Significant differences in the ANOVA were detected for the 10 metabolites. The anthocyanins Del_G, Pet_G, and Mal_G were present at significantly higher concentrations in the black group, although they showed high standard deviation values because some black lines fall short of this group of anthocyanins. The anthocyanin Cya_G was significantly higher in black and red groups, but was also absent in some lines of these color groups. The anthocyanin Pel_G also showed significantly higher concentrations in the red group, but was also absent in some red-seeded lines. Concerning flavonols, Myr_G and Que_G showed significantly higher concentrations in the black group and Que_GAc in the brown one. Kae_G and Kae_GAcII showed higher values in both the red and yellow groups.

**Table 2:** Mean values (in µg/g ± standard deviation) according to seed coat color classes for 5 anthocyanins and 5 flavonols. Analysis of variance (ANOVA) and Tukey test were conducted to detect significant differences between color classes. ***, *p* < 0.001

Trait	black	red	brown	yellow	cream	white	*F* value (p)
Del_G	290.8 ± 204.8 b	1.1 ± 3.2 a	2.4 ± 8.3 a	0.5 ± 1.9 a	1.8 ± 5.0 a	0.0 ± 0.0 a	87.7***
Pet_G	55.0 ± 71.4 b	0.0 ± 0.0 a	0.0 ± 0.0 a	0.0 ± 0.0 a	0.2 ± 1.1 a	0.0 ± 0.0 a	26.0***
Mal_G	16.7 ± 29.0 b	0.3 ± 1.7 a	0.2 ± 1.4 a	0.0 ± 0.0 a	0.0 ± 0.0 a	0.0 ± 0.0 a	14.4***
Cya_G	37.7 ± 82.5 c	28.5 ± 26.8 bc	7.7 ± 7.8 ab	1.8 ± 3.4ab	3.1 ± 4.9 a	0.0 ± 0.0 a	9.5***
Pel_G	17.3 ± 38.1 b	43.9 ± 49.0 c	4.2 ± 8.6 ab	1.3 ± 3.0 ab	2.8 ± 6.0 a	0.0 ± 0.0 a	19.8***
Kae_G	96.0 ± 189.5 ab	458.8 ± 555.1 c	137 ± 191.7 ab	707.3 ± 564.5 c	229.4 ± 425.7 b	0.0 ± 0.0 a	19.3***
Kae_GAcII	21.1 ± 30.5 ab	131.5 ± 139.7 e	76.1 ± 72.8 cd	134.2 ± 58.0 de	44.3 ± 72.8 bc	0.0 ± 0.1 a	25.6***
Myr_G	89.8 ± 88.8 b	0.5 ± 0.8 a	0.5 ± 1.1 a	0.1 ± 0.2 a	0.4 ± 1.1 a	0.0 ± 0.0 a	44.7***
Que_G	47.1 ± 51.4 d	32.4 ± 36.1 cd	26.4 ± 24.8 bc	9.2 ± 7.0 ab	8.5 ± 15.2 a	0.0 ± 0.0 a	21.1***
Que_GAc	5.8 ± 4.9 ab	8.4 ± 7.9 b	17.9 ± 26.2 c	2.8 ± 2.2 ab	2.3 ± 4.1 ab	0.0 ± 0.1 a	16.1***

Different phenolic profiles were distinguished within black-seeded lines in the SDP:

i) lines that presented exclusively the three anthocyanins related with blue-dark blue colors, Del_G, Pet_G, and Mal_G. This group included the line SDP294 derived from the well-known anthracnose differential cultivar TU [34].
ii) lines with only two anthocyanins related with blue-dark blue colors, Del_G, and Pet_G. This group included the line SDP225 derived from the black anthracnose differential cultivar Cornell49242 [34].
iii) lines with the anthocyanin Del_G related with blue colors but also Cya_G and Pel_G related with red-pink-orange colors;
iv) two lines (SDP099 and SDP124) showed only the anthocyanins Cya_G and Pel_G related with red-pink-orange colors.

In general, the dominant phenolic profile within the red-seeded lines showed high levels of the anthocyanins, Cya-G and Pel_G. Only two red-seeded lines, SBP256 (a line derived from the anthracnose differential cultivar MDRK; [34]) and SBP281, lack anthocyanins but showed high levels of the Que_G flavonol. The Kae_G flavonol was the most abundant within yellow and cream seeds.

### GWAS

Based on the QQ-plots obtained (Additional files 2, 3) the FASTmrEMMA model fitted the data better so was chosen as the model to be used in the GWAS. A total of 31 significant SNP-trait associations (QTNs) were identified for all traits except Pet_G, seed color Cream-NC, and Red_NR (Table 3). Four of the QTNs were associated with more than one trait: SNP04_161102 was associated with Myr_G and Del_G; SNP07_28996339 was associated with color traits L* and White_NW; SNP08_3066515 was associated with Que_G, Kae_G, Kae_GAcII and with the color trait Pattern_NP; and SNP08_3694747 was associated with Cya_G and Pel_G. The QTNs identified were located in chromosomes Pv01, Pv02, Pv04, Pv06, Pv07, Pv08, Pv09, and Pv10. The chromosome Pv08 includes the largest number of QTNs (nine), among which seven are associated with phenolic metabolites and two are associated with seed color traits. All the QTNs identified in chromosomes Pv06 and Pv07 (three and six QTNs, respectively) were associated with seed color traits.

**Table 3:**
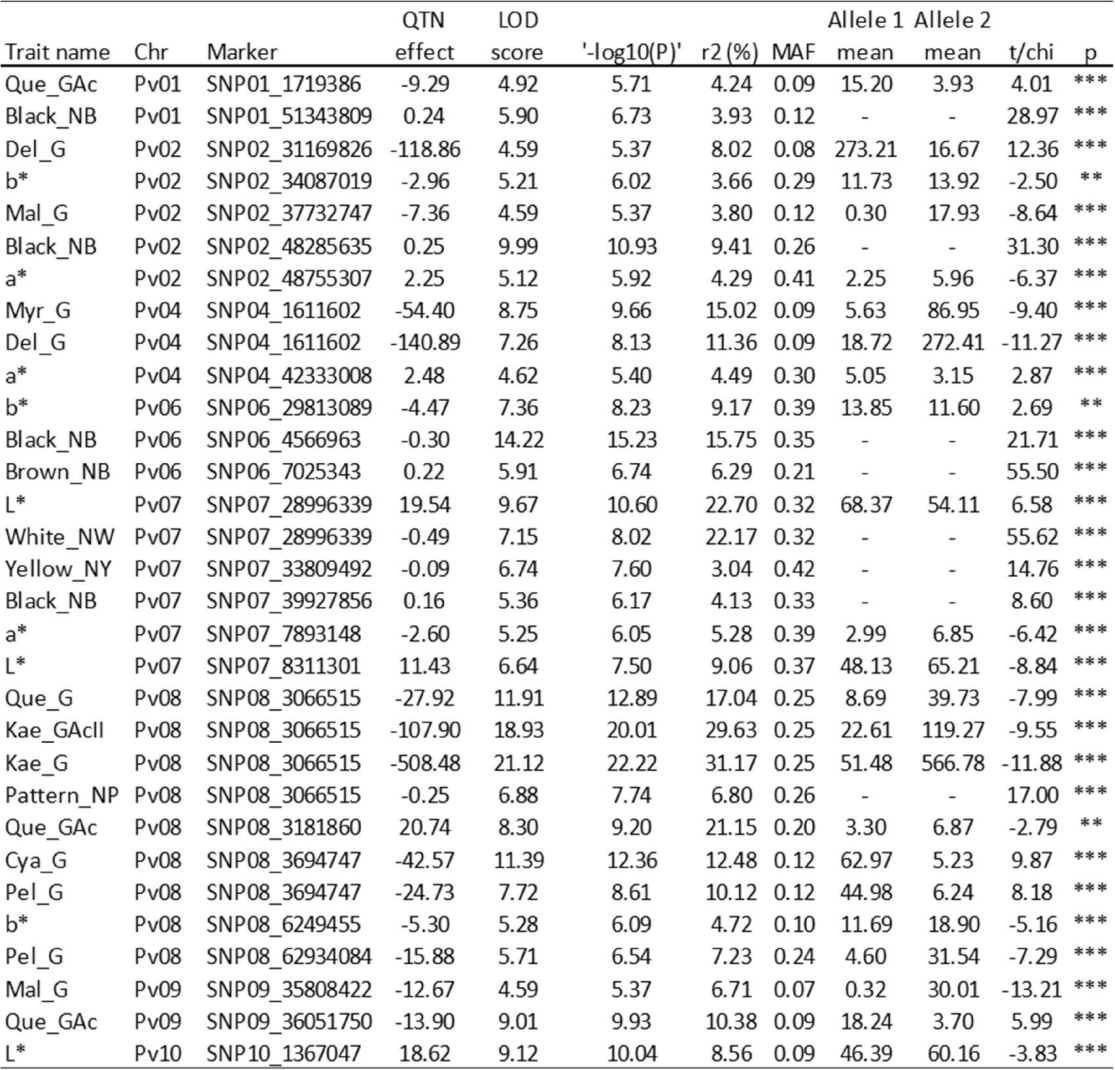
Significant associations (-log(p) ≥ 5.4) identified for each trait using the FASTmrEMMA method. T-student or Chi-square tests were used to investigate the significant differences between each trait and the two alleles of the associated SNP. *, *p* < 0.05; **, 0.01 < *p* > 0.001; ***, *p* < 0.001

**Fig. 2:**
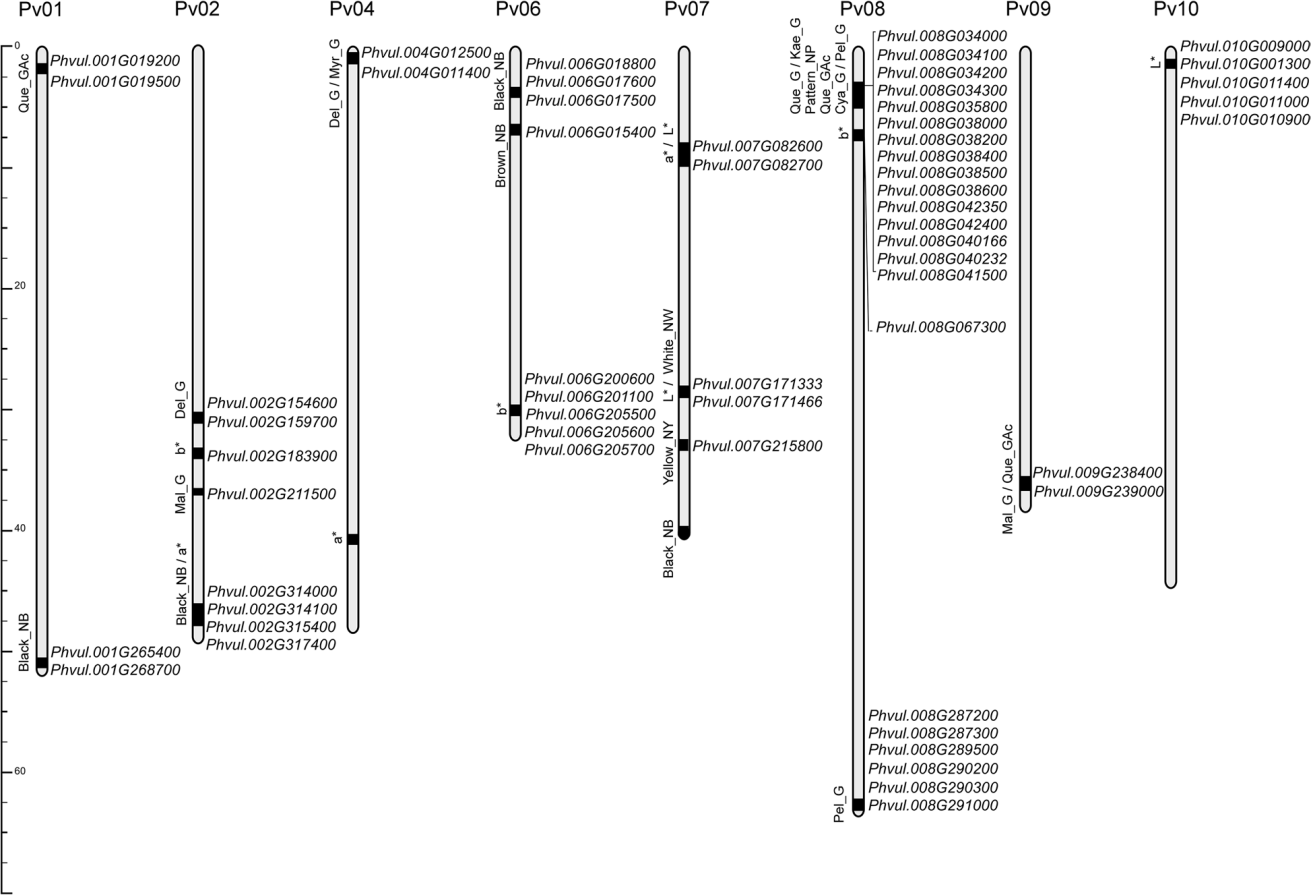
Chromosome regions associated with seed coat phenolic metabolites and/or seed coat colors in the Spanish Diversity Panel. Tentative candidate genes identified in each region are indicated on the right

Considering a 350‐Kb window centered for each significant SNP, these 31 QTNs were grouped in 20 QTL (Table 4; Fig. 2): 6 for phenolic metabolites (Phe), 13 for seed coat color (C), and 1 including both types of traits (Phe/C).

**Table 4:** QTL identified for phenolic metabolites and color traits in the Spanish diversity panel. Na/Nc: number of annotated/candidate genes

QTL	Chr	SNP	Trait/s	Position (Mbp)	Na/Nc
Phe_Pv01(1.3)	Pv01	SNP01_1719386	Que_GAc	1.36..2.06	69/2
C_Pv01(50.9)	Pv01	SNP01_51343809	Black_NB	50.99..51.69	69/2
Phe_Pv02(30.8)	Pv02	SNP02_31169826	Del_G	30.81..31.51	65/2
C_Pv02(33.7)	Pv02	SNP02_34087019	b	33.73..34.43	63/1
Phe_Pv02(37.3)	Pv02	SNP02_37732747	Mal_G	37.8..38.08	55/1
C_Pv02(47.9)	Pv02	SNP02_48285635	Black_NB	47.93..49.10	146/4
	Pv02	SNP02_48755307	a		
Phe_Pv04(1.2)	Pv04	SNP04_1611602	Del_G, Myr_G	1.26..1.96	69/2
C_Pv04(41.9)	Pv04	SNP04_42333008	a	41.98..42.68	57/0
C_Pv06(4.2)	Pv06	SNP06_4566963	Black_NB	4.21..4.91	22/3
C_Pv06(6.6)	Pv06	SNP06_7025343	Brown_NB	6.67..7.37	17/1
C_Pv06(29.4)	Pv06	SNP06_29813089	b	29.46..30.16	104/5
C_Pv07(7.5)	Pv07	SNP07_7893148	a	7.54..8.66	71/2
	Pv07	SNP07_8311301	L		
C_Pv07(28.6)	Pv07	SNP07_28996339	L, White_NW	28.64..29.34	50/2
C_Pv07(33.4)	Pv07	SNP07_33809492	Yellow_NY	33.45..34.15	65/1
C_Pv07(39.5)	Pv07	SNP07_39927856	Black_NB	39.57..40.04	61/0
Phe/C_Pv08(2.7)	Pv08	SNP08_3066515	Que_G, Kae_G, Pattern_NP	2.71..4.04	139/15
	Pv08	SNP08_3181860	Que_Gac		
	Pv08	SNP08_3694747	Cya_G, Pel_G		
C_Pv08(5.8)	Pv08	SNP08_6249455	b	5.89..6.59	77/1
Phe_Pv08(62.5)	Pv08	SNP08_62934084	Pel_G	62.58..63.28	70/6
Phe_Pv09(35.4)	Pv09	SNP09_35808422	Mal_G	35.45..36.40	71/2
		SNP09_36051750	Que_GAc,		
C_Pv10(1.0)	Pv10	SNP10_1367047	L	1.01..1.71	51/5

### Putative candidate genes

A total of 1391 genes were annotated underlying the 20 QTL, 165 of which have no annotated function. Candidate genes were tentatively proposed based on three criteria described in the Material and Methods section. No candidate genes were identified for C_Pv04(41.9), and C_Pv07(39.5). In all, 58 genes were proposed as candidate genes (Table 5; Fig. 2):

**Table 5:** Tentative candidate genes identified to be involved in the phenylpropanoid pathway in common bean. According to the *Arabidopsis* phenylpropanoid pathway genes identify, a classification of each gene in structural, regulator or transport gene is suggested

QTL	Candidate gene	Function associated (www.phytozome.net)	Pathway
Phe_Pv01(1.3)	*Phvul.001G019200*	MYB-like dna-binding protein MYB	Regulatory
	*Phvul.001G019500*	serine protease family s10 serine carboxypeptidase	Structural
C_Pv01(50.9)	*Phvul.001G265400*	crocetin glucosyltransferase / ugt75l6	Structural
	*Phvul.001G268700*	sf98 – O-methyltransferase family protein	Structural
Phe_Pv02(30.8)	*Phvul.002G154600*	mate efflux family protein	Transport
	*Phvul.002G159700*	myb-like DNA-binding protein myb	Regulatory
C_Pv02(33.7)	*Phvul.002G183900*	oxidoreductase, 2og-Fe II oxygenase family protein	Structural
Phe_Pv02(37.3)	*Phvul.002G211500*	oxidoreductase, 2og-Fe II oxygenase family protein	Structural
C_Pv02(47.9)	*Phvul.002G314000*	coniferyl-aldehyde dehydrogenase (ref1)	Structural
	*Phvul.002G314100*	coniferyl-aldehyde dehydrogenase (ref1)	Structural
	*Phvul.002G315400*	cytochrome p450 cyp2 subfamily	Structural
	*Phvul.002G317400*	Cation transporting ATPase, C-terminus (Cation_ATPase_C)	Transport
Phe_Pv04(1.2)	*Phvul.004G012500*	cinnamoyl coa reductase-like protein	Structural
	*Phvul.004G011400*	MYB domain protein 119	Regulatory
C_Pv06(4.2)	*Phvul.006G018800*	flavonoid 3’,5’-hydroxylase (cyp75a)	Structural
	*Phvul.006G017600*	UDP-glycosyltransferase 85A1-related	Structural
	*Phvul.006G017500*	UDP-glycosyltransferase 85A1-related	Structural
C_Pv06(6.6)	*Phvul.006G015400*	flavonoid 3’,5’-hydroxylase (cyp75a)	Structural
C_Pv06(29.4)	*Phvul.006G200600*	alcohol dehydrogenase related	Structural
	*Phvul.006G201100*	flavonol 3-o-glucosyltransferase	Structural
	*Phvul.006G205500*	glucosyl/glucuronosyl transferases	Structural
	*Phvul.006G205600*	glucosyl/glucuronosyl transferases	Structural
	*Phvul.006G205700*	glucosyl/glucuronosyl transferases	Structural
C_Pv07(7.5)	*Phvul.007G082600*	peroxidase / lactoperoxidase	Structural
	*Phvul.007G082700*	peroxidase / lactoperoxidase	Structural
C_Pv07(28.6)	*Phvul.007G171333*	transcription factor tt8 (*P* locus)	Regulatory
	*Phvul.007G171466*	*P* locus	Regulatory
C_Pv07(33.4)	*Phvul.007G215800*	myb transcription factor	Regulatory
Phe/C_Pv08(2.7)	*Phvul.008G034000*	cyanohydrin beta-glucosyltransferase	Structural
	*Phvul.008G034100*	glucosyl/glucuronosyl transferases	Structural
	*Phvul.008G034200*	glucosyl/glucuronosyl transferases	Structural
	*Phvul.008G034300*	glucosyl/glucuronosyl transferases	Structural
	*Phvul.008G035800*	cyanohydrin beta-glucosyltransferase	Structural
	*Phvul.008G038000*	myb-like dna-binding protein	Regulatory
	*Phvul.008G038200*	transcription factor myb113-related	Regulatory
	*Phvul.008G038400*	transcription factor myb113-related	Regulatory
	*Phvul.008G038500*	transcription factor myb113-related	Regulatory
	*Phvul.008G038600*	transcription factor myb113-related	Regulatory
	*Phvul.008G042350*	isoflavone-7-o-beta-glucoside 6’’-o-malonyltransferase	Structural
	*Phvul.008G042400*	isoflavone-7-o-beta-glucoside 6’’-o-malonyltransferase	Structural
	*Phvul.008G040166*	Isoliquiritigenin 2’-O-methyltransferase	Structural
	*Phvul.008G040232*	Isoliquiritigenin 2’-O-methyltransferase	Structural
	*Phvul.008G041500*	MYB-like dna-binding protein MYB	Regulatory
C_Pv08(5.8)	*Phvul.008G067300*	MYB-like dna-binding protein MYB	Regulatory
Phe_Pv08(62.5)	*Phvul.008G287200*	aldo/keto reductase // s ubfamily not named	Structural
	*Phvul.008G287300*	aldo/keto reductase // subfamily not named	Structural
	*Phvul.008G289500*	phenylalanine ammonia-lyase	Structural
	*Phvul.008G290200*	UDP-glycosyltransferase 71c4	Structural
	*Phvul.008G290300*	UDP-glycosyltransferase 71c4	Structural
	*Phvul.008G291000*	UDP-glycosyltransferase 82A1	Structural
Phe_Pv09(35.4)	*Phvul.009G238400*	peroxidase 16-related	Structural
	*Phvul.009G239000*	h + -transporting ATPase	Transport
C_Pv10(1.0)	*Phvul.010G009000*	glucosyl/glucuronosyl transferases	Structural
	*Phvul.010G001300*	glucosyl/glucuronosyl transferases	Structural
	*Phvul.010G011400*	serine carboxypeptidase-like 1-related	Structural
	*Phvul.010G011000*	cytochrome p450 71b21-related	Structural
	*Phvul.010G010900*	cytochrome p450 71b21-related	Structural

#### QTL Phe_Pv01(1.3)

The flavonol Que_GAc was associated with this region. The genes *Phvul.001G019200,* and *Phvul.001G019500* showed high homology with *Arabidopsis* proteins (Additional file 5). *Phvul.001G019500* showed high homology with NP_179883.1, an enzyme sinapoyl-glucose: anthocyanin acyltransferase required for the synthesis of the sinapoylated anthocyanins. *Phvul.001G019200* showed high homology with the *MYB* domain protein NP_195574.1 with a regulatory function in the phenylpropanoid pathway.

#### QTL C_Pv01(50.9)

Black seed color was associated with this region, in which two candidate genes were considered. The *Phvul.001G265400* gene encodes a glucosyltransferase that showed high homology with protein NP_193146.1 (Additional file 5), an anthocyanin 5-O-glucosyltransferase involved in the phenylpropanoid pathway in *Arabidopsis*. The gene *Phvul.001G268700* encodes an O-methyltransferase family protein showing high homology with protein NP_200227.1 (Additional file 5), a flavonol 3-O-methyltransferase that is highly active towards quercetin and myricetin [35].

#### QTL Phe_Pv02(30.8)

The anthocyanin Del_G was associated with this region. The gene *Phvul.002G154600* encodes a mate efflux family protein and showed high homology with protein NP_191462.1 (Additional file 5), the MATE transporter, TT12, which acts as a vacuolar flavonoid/H + -antiporter active in proanthocyanidin accumulating cells of the seed coat in *Arabidopsis*. Gene *Phvul.002G159700* codifies an *MYB-like* protein and shows high homology with protein NP_199744.1 (Additional file 5), which is and *MYB111* implicated in the regulation of the flavonol biosynthetic process.

#### QTL C_Pv02(33.7)

The color vector b* was located in this QTL. The gene *Phvul.002G183900* could be considered a candidate gene, showing high homology with proteins NP_001190266.1 and NP_201164.1 (Additional file 5), two flavonol synthases that catalyze the formation of flavonols from dihydroflavonols.

#### QTL Phe_P02(37.3)

The anthocyanin Mal_G was located in this QTL. The gene *Phvul.002G211500* showing high homology with flavonol synthases NP_001190266.1, and NP_201164.1 of *Arabidopsis* (Additional file 5) can be considered the candidate.

#### QTL C_Pv02(47.9)

The seed color trait Black_NB and the vector a* were located in this region. Of the four candidate genes identified, *Phvul.002G314000* and *Phvul.002G314100* are involved in the phenylpropanoid pathway. The gene *Phvul.002G315400* codifies a protein of the cytochrome p450 cyp2 subfamily and shows high homology with protein NP_196416.1 (Additional file 5), which is required for flavonoid 3’ hydroxylase (F3´H) activity in *Arabidopsis*. *Phvul.002G317400* showed high homology with protein NP_173169.2 (Additional file 5), a plasma H + -ATPase required for vacuolar deposition of proanthocyanidins in *Arabidopsis* seeds.

#### QTL Phe_Pv04(1.2)

The anthocyanin Del_G and the flavonol Myr_G were located in this region. Two candidate genes were identified based on their homology with *Arabidopsis* genes (Additional file 5): *Phvul.004G011400* showed high homology with the *MYB* regulatory protein, NP_195574.1, whereas *Phvul.004G012500* showed high homology with NP_199094.1, a dihydroflavonol reductase that is the first enzyme leading to the production of anthocyanins [36].

#### QTL C_Pv06(4.2)

Seed color trait Black_NB was located in this region. *Phvul.006G018800* encodes a flavonoid 3’,5’-hydroxylase (F3’,5’H) with high homology with protein NP_196416.1 (Additional file 5), a cytochrome P450 superfamily protein required for F3’H activity in *Arabidopsis*. This enzyme abundance relative to chalcone synthase determines the quercetin/kaempferol metabolite ratio in *Arabidopsis*. Genes *Phvul.006G017600* and *Phvul.006G017500* codify glucosyltransferases and showed high homology with the protein NP_197207.1, an anthocyanidin 3-O-glucosyltransferase which specifically glucosylates the 3-position of the flavonoid C-ring.

#### QTL C_Pv06(6.6)

The seed color trait Brown_NB was located in this region. One candidate gene was identified, *Phvul.006G015400,* that encodes a F3’,5’H involved in the anthocyanin and pigment biosynthesis pathways. This gene showed high homology with protein NP_196416.1 (Additional file 5) required for F3’H activity in *Arabidopsis.*

#### QTL C_Pv06(29.4)

The seed color vector b* was located in this region. The candidate gene *Phvul.006G200600* participates in phenylpropanoid biosynthesis. *Phvul.006G201100* encodes a flavonol 3-O-glucosyltransferase that has an essential role in the biosynthesis of anthocyanins, being involved in the formation of delphinidin 3-O-glucoside, pelargonidin 3-O-glucoside, kaempferol triglucoside, and myricetin*. Phvul.006G201100* and the three genes organized in tandem, *Phvul.006G205500*, *Phvul.006G205600*, and *Phvul.006G205700* showed high homology with NP_197207.1 (Additional file 5), a UDP-glucosyl transferase 78D2 involved in the phenylpropanoid pathway in *Arabidopsis*.

#### QTL C_Pv07(7.5)

Color vectors L* and a* were associated with this region. Two candidate genes organized in tandem, *Phvul.007G082600* and *Phvul.007G082700,* codify a peroxidase involved in the phenylpropanoid biosynthesis.

#### QTL C_Pv07(28.6)

The color trait White_NW and the color vector L* were associated with this region. The two candidate genes proposed for the *P* gene, *Phvul.007G171333* and *Phvul.007G171466* [21], were included in this QTL. *Phvul.007G171333* gene showed high homology with NP_192720.2 (Additional file 5), a regulation factor of flavonoid pathways in *Arabidopsis*.

#### QTL C_Pv07(33.4)

The color trait Yellow_NY was associated with this region. One candidate gene, *Phvul.007G215800* coding an *MYB* transcription factor, was identified. This gene shows high homology with the *Arabidopsis* protein NP_195574.1 (Additional file 5), *MYB111* that is a member of the R2R3-MYB transcription factor that regulates flavonol accumulation.

#### QTL Phe/C_Pv08(2.7)

This was the major QTL identified in this work, including two anthocyanins, Cya_G and Pel_G, three flavonols, Que_G, Que_Gac, and Kae_G, and the color trait Pattern_NP. A total of 15 candidate genes were considered, most of them showing high homology with *Arabidopsis* proteins involved in the phenylpropanoid pathway (Additional file 5). The genes *Phvul.008G034000, Phvul.008G034100*, *Phvul.008G034200*, *Phvul.008G034300,* and *Phvul.008G035800* codify glucosyltransferases, a type of enzyme that acts on a wide range of substrates including phenolics. The genes *Phvul.008G038000*, *Phvul.008G038200*, *Phvul.008G038400*, *Phvul.008G038500*, *Phvul.008G038600,* and *Phvul.008G041500* encode *MYB* transcription factors involved in the regulation of phenylpropanoid biosynthesis. The two genes *Phvul.008G040166* and *Phvul.008G040232* encode an isoliquiritigenin 2’-O-methyltransferase, an enzyme involved in the phenylpropanoid pathway. The two genes *Phvul.008G042350*, and *Phvul.008G042400* encode an enzyme isoflavone-7-O-beta-glucoside 6’’-O-malonyltransferase, which is implicated in the flavonoid biosynthesis pathway.

#### QTL C_Pv08(5.8)

The color vector b* was associated with this QTL. One candidate gene, *Phvul.009G067300* showed high homology (Additional file 5) with the *MYB* protein NP_195574.1 involved in the regulation of phenylpropanoid biosynthesis in *Arabidopsis*.

#### QTL Phe_Pv08(62.5)

The anthocyanin Pel_G was associated with this QTL. Two genes organized in tandem, *Phvul.008G287200* and *Phvul.008G287300*, were considered candidate genes as they are part of flavonoid biosynthesis. Gene *Phvul.008G289500* encodes a phenylalanine ammonia-lyase, an important plant enzyme that eliminates ammonia from phenylalanine to form trans-cinnamic acid, a precursor of flavonoids. Genes *Phvul.008G290200* and *Phvul.008G290300,* and *Phvul.008G291000* codify UDP-glucosyltransferases and showed high homology with *Arabidopsis* proteins NP_200217.1 and NP_193146.1 (Additional file 5) that are involved in the phenylpropanoid pathway.

#### QTL Phe_Pv09(35.4)

The anthocyanin Mal_G and the flavonol Que_GAc were associated with this QTL. Two candidate genes were identified: gene *Phvul.009G238400* which encodes a peroxidase involved in phenylpropanoid biosynthesis, and *Phvul.009G239000,* which codes an H + -transporting ATPase with high homology with the *Arabidopsis* protein NP_173169.2 (Additional file 5), that is required for the formation of proanthocyanidins in the seed coat endothelium and their transport from the cytosol to the vacuole.

#### QTL C_Pv10(1.0)

The color vector L* was associated with this QTL. Two genes organized in tandem, *Phvul.010G009000* and *Phvul.010G001300*, showed high homology with the UDP-glucosyltransferase 78D2 protein NP_197207.1 (Additional file 5). The function assigned to *Phvul.010G009000* is a glucosyl/glucoronosyl transferase, but no associated function was assigned to the gene *Phvul.010G001300*. *Phvul.010G011400* codifies a serine carboxypeptidase-like protein and could be considered a candidate gene because it shows high homology with the protein NP_179883.1 (Additional file 5), which encodes a sinapoyl glucose:anthocyanin acyltransferase, an enzyme required for the synthesis of the sinapoylated anthocyanins. Two other candidate genes organized in tandem can be considered, *Phvul.010G011000* and *Phvul.010G010900*, both of which codify a cytochrome p450 71b21. These genes showed high homology with *Arabidopsis* protein NP_196416.1 (Additional file 5) that is required for F3’H activity.

## Discussion

The GWAS conducted in this work led to the identification of 20 genomic regions or QTL associated with the synthesis of 10 flavonoids (5 anthocyanins and 5 flavonols) and with 10 seed coat color traits using a set of 308 common bean lines of the SDP. Six QTL were identified for phenolic metabolites (Phe) on chromosomes Pv01, Pv02, Pv04, Pv08, and Pv09; 13 for seed coat color (C) on chromosomes Pv01, Pv02, Pv06, Pv07, and Pv10, and one QTL including both types of traits (Phe/C) located on chromosome Pv08. However, no candidate genes or relationship with the phenylpropanoid pathway had been identified for the QTLs C_Pv04(41.9), and C_Pv07(39.5).

The longest QTL identified was Phe/C_Pv08(2.7), ranging from 2.71 to 4.04 Mbp of chromosome Pv08. This QTL was the only one associated with both phenolic metabolites (the three flavonols Que_G, Que_GAc, and Kae_G, the two anthocyanins Cya_G and Pel_G) and a seed color trait (pattern-no pattern). At this position of chromosome Pv08, the complex *C* locus was described to be flanked by the markers OW17S^600^, and OAP2^700^, whose estimated physical positions were 3.2 and 9.6 Mbp, respectively [18, 19]. The *C* locus is a complex group of tightly linked genes involved in the determination of seed coat color and color pattern [18]: *C* (sulfur-white or primrose-yellow seed coat), *R* (dominant red color gene), *M* (controlling mottled pattern; [37]), *Pr* (related to red color in the dark pattern color zones; [17]), and *Gy* (controlling the change from pale greenish-yellow to strong greenish-yellow seed coat color; [38])*.* Even several alleles of these genes have been proposed for the different patterning of coat pigments, such as the *C*^*st*^ allele that causes striping on seed coats and pods [39]. Recently, the seed pattern “corona color” was associated with the SNP Chr08pos3300438 through a GWAS conducted in a collection of 295 yellow common bean genotypes [40], whose physical position is very close to that of the QTL Phe/C_Pv08(2.7). In the present work, a total of 15 candidate genes have been proposed for this QTL, 9 of which are structural genes encoding biosynthetic enzymes involved in the phenylpropanoid pathway, and 6 probably have regulatory functions in this pathway. These regulatory genes include a *MYB* transcription factor that forms part of the MBW (MYB-βHLH-WD40) ternary complex, which activates the late biosynthetic proteins required for the anthocyanin and proanthocyanidin synthesis in other species [41]. One of these genes encoding a *MYB113-*related factor, *Phvul.008G038400*, has been proposed as a gene responsible for the black seed coat color expression in the cultivar TU [26]. Another wide QTL for phenolic metabolites, Phe_Pv08(62.5), was located at the end position of chromosome Pv08, between 62.58–63.28 Mbp, associated with the synthesis of the anthocyanin Pel_G. Six candidate genes have been proposed for this QTL, all being structural genes. A recent study localized a QTL associated with the trait `post-processing color retention` in black seeds at chromosome Pv08 using two RIL populations [42], which supports the importance of this chromosome for dry common bean pigmentation and the phenylpropanoid pathway.

The five remaining QTL involved in the synthesis of phenolic metabolites were located at chromosomes Pv01 [Phe_Pv01(1.3)], Pv02 [Phe_Pv02(30.8), Phe_Pv02(37.3)], Pv04 [Phe_Pv04(1.2)], and Pv09 [Phe_Pv09(35.4)]. For these QTL, 9 candidate genes have been proposed, 4 of which were structural genes, three were regulatory, and two were involved in the anthocyanin transport. Of special interest are the putative transport genes identified, *Phvul.002G154600* and *Phvul.009G239000*, that underlie the QTL Phe_Pv02(30.8) and Phe_Pv09(35.4), both of which are associated with the anthocyanin Mal_G. These genes show high homology with the *Arabidopsis* proteins NP_191462.1 and NP_173169.2, respectively, both of which are involved in the transportation of proanthocyanidin precursors into the vacuoles of the seed coat endothelium [43, 44]. This is the first work that relates the common bean genes *Phvul.002G154600* and *Phvul.009G239000* with the phenylpropanoid pathway, in particular with the transport of the anthocyanin Mal_G.

Concerning seed coat color, a significant and negative association was observed between the color vector L* and the 10 phenolic metabolites. This result is expected as L* detects the brightness from 0 (black) to 100 (white), and anthocyanins and flavonols are not present in white seed coats. However, L* values within white-seeded lines range from 72.38 to 86.17, suggesting that white color shows different intensities among genotypes. This result agrees with the multiple recessive alleles responsible for white seed color identified for the *P* locus, a major switch in the flavonoid biosynthetic pathway with recessive alleles blocking the expression of other color genes. Probably, the multiple recessive alleles responsible for white seed color at the *P* locus [21] have a phenotypic effect on the expression of the L* color vector. The *P* locus was located at 28,75–28,77 Mbp of chromosome Pv07 [21], underlying the QTL C_Pv07(28.6) identified for white-NW color and L*. Accordingly, other authors also located the L* seed color vector at this position, associated with the SNP Chr07pos29169848 [40]. In the present work, two other regions were identified for the L* vector: C_Pv07(7.5) and C_Pv10(1.0). C_Pv07(7.5) could correspond with the *Asp* gene mapped at the beginning of chromosome Pv07 [45, 46], a gene responsible for the shiny seed coat, which in recessive genotypes displays a dull coat [47]. It has been demonstrated that recessive *asp* genotypes show 19% less of the total anthocyanin content compared with *Asp* [24]. This could explain why color vector a* is also located at C_Pv07(7.5), since positive values for a* measure red color intensity and hence are related to anthocyanin content. The QTN a*- SNP07_7893148, located at C_Pv07(7.5), showed a negative effect (-2,60; Table 3) indicating reduced a* values and less anthocyanin content. Two candidate genes within this QTL, *Phvul.007G082600* and *Phvul.007G082700*, involved in the phenylpropanoid biosynthesis, could be the genes responsible for a* and L* values. Recently, Sadohara et al. [40] also identified an association between the seed coat color vector a* and the SNP Chr07pos634407, whose position corresponds with that of C_Pv07(7.5). Another association identified by Sahodara et al. [40] between a*- Chr04pos41620411 was also identified in this work at C_Pv04(41.9), although no candidate genes were proposed for this QTL.

Different types of phenolic profiles can be distinguished within black-seeded lines in the SDP, suggesting that black color can be obtained from the accumulation of different types of anthocyanins, which was previously observed by Rodríguez Madrera et al. [33]. Most of the black seed coat phenotypes analyzed in this work are characterized by a significantly higher concentration of delphinidin-based anthocyanins (Del_G, Pet_G, Mal_G) which was also previously observed by other authors [7, 48]. The existence of different phenolic profiles within black-seeded lines agrees with the four QTL identified for the black_NB seed coat: C_Pv01(50.9), C_Pv02(47.9), C_Pv06(4.2), and C_Pv07(39.5). The QTL C_Pv01(50.9) may correspond with the association identified by Sadohara et al. [40] between the L* color vector and the SNP Chr01pos50086405, considering that L* detects the brightness from 0 (black) to 100 (white). This QTL could be related to the *Co-1* anthracnose resistance cluster located at this position of chromosome Pv01 [49], as polyphenols play an important role in plant defense mechanisms against diseases and pests [50]. Another QTL related with the black seed trait, C_Pv02(47.9), is closely located to the *I* gene (candidate gene *Phvul.002G324100*; [51]) conferring resistance against several common mosaic potyviruses [51–54]. The *I* gene is known to be closely linked to the *B* color gene on Pv02, with purple seed color genotypes being identified as highly resistant to the common bean common mosaic virus [55]. Although no associations have been identified for red_NR color in this work, the *Rk* gene involved in the synthesis of red or garnet brown seed colors, for which a series of four alleles have been described, was linked to the *B* gene in Pv02 [56]. The QTL C_Pv02(47.9) is associated with the black seed trait but also with the a* vector, which represents colors from green (negative values) to red (positive values), so this color vector is likely to be related to the *Rk* gene. Five candidate genes have been proposed for these QTL, three of them previously associated with the phenylpropanoid pathway in common bean, and two proposed in this work based on the association mapping and the homology analysis with *Arabidopsis*: *Phvul.002G315400* and *Phvul.002G317400*.

The other QTL including the black_NB seed trait, C_Pv06(4.2), corresponds with the *V* color gene. The gene *Phvul.006G018800* located in this region encodes a F3’,5’H and was previously proposed as the gene controlling the black seed coat color in the cultivar TU [26]. Later, McClean et al. [27] confirmed that *V* encodes an F3’,5’H required for the expression of dihydromyricetin-derived metabolites. Beninger et al. [6, 24] affirmed that anthocyanin production is dependent on the *V* gene, since the presence of dominant genotypes *V* leads to anthocyanin accumulation, whereas recessive genotypes *v* results in flavonol accumulation. In common bean, only two genes encoding an F3’,5’H have been identified, one is this gene *Phvul.006G018800* that corresponds with the *V* gene located at the QTL C_Pv06(4.2) and is associated with black seed colors. The other, *Phvul.006G015400*, is located at the QTL C_Pv06(6.6) identified in this work associated with brown seed colors.

## Conclusions

Results obtained provide new data on the complex network of genes involved in the genetic control of phenolic compounds and seed color in common bean. Chromosome Pv08 was identified as the main chromosome involved in the phenylpropanoid pathway and in consequence, in the common bean seed pigmentation, involving three regions, Phe/C_Pv08(2.7), C_Pv08(5.8), and Phe_Pv08(62.5). Candidate genes have been tentatively proposed for color genes *B* and *Rk* on Pv02, *Asp* on Pv07, and complex* C* on Pv08. Candidate genes previously proposed by other authors for the color genes *V* and *P* have been validated in this GWAS. This work opens the opportunity for future gene validations and can be useful for a targeted breeding of phenolic profiles of legume seeds, which is of relevance for both nutritional qualities and the plant resistance or tolerance to pests and diseases.

## Material and methods

### Plant material

The SDP constituted of 308 homozygous lines [32] was used in this study. The SDP includes lines derived from 220 landraces, mostly from the updated Spanish Core Collection [45]; from 51 elite cultivars, mostly cultivated in Europe for snap consumption; and from 37 lines representing traditional old cultivars and well-known breeding lines. All lines were grown in the same season in 2018 (43◦ 29′01́́ ‘N, 5◦ 26′11’W; elevation 6.5 m) using a randomized complete block design with one replicate per line consisting of ten plants distributed in a 1 m row plot. A subsample of 11 lines randomly selected were grown in 2019 using the same experimental conditions as in 2018. Dry seeds were maintained in controlled conditions (-20ºC under vacuum) until their chemical analysis.

### Seed coat phenolic profile

SDP lines were characterized for their seed extractable phenolic profiles corresponding to 13 anthocyanins derived from delphinidin, cyanidin, petunidin, pelargonidin, and malvidin aglycones and 15 flavonols derived from kaempferol, myricetin, and quercetin according with our previous work ([33]; Table 1). To confirm the stability of the characterization, a subsample of 11 lines was grown and reanalyzed in 2019 and the data compared with the 2018 characterization by analysis of variance (ANOVA) using the R Project for Statistical Computing [57].

### Seed coat color

Seed coat color was quantitatively characterized using a Minolta CM-2300d Spectrophotometer, (Konica Minolta Inc., Madrid, Spain) to measure three vectors in the CIELAB scale: L* detects the brightness from 0 (black) to 100 (white), a* represents color from green (negative values) to red (positive values), and the b* measures blue (negative values) to yellow (positive values). Seed coat color was measured in three seeds per line, and the least-squares mean (LSmeans) value for L*, a*, and b* was obtained using the “lsmeans” package in R [57]. Histograms showing the distribution of each qualitative trait were obtained using the rMVP package [58] in R. Spearman´s correlation coefficients between the metabolites analyzed and the color vectors were calculated in R [57].

The main seed coat color was also qualitatively characterized as white-no white, black-no black, brown-no brown, yellow-no yellow, cream-no cream, and red-no red. The color patterns were characterized as the absence or presence of patterns corresponding to the descriptors, partly colored, mottled, or color in the hilum ring. An ANOVA and a Tukey test were conducted in R [57] to significant differences in phenolic metabolite contents and color vectors L*, a*, and b* among the seed color groups.

### Genotyping

Genotyping-by-sequencing (GBS), as described by [59], was carried out at BGI-Tech (Copenhagen, Denmark) using the *ApeKI* restriction enzyme. The sequencing reads from different genotypes were aligned [26] using the reference genome v2.1 [60, 61]. Data were filtered in Tassel v5.2 software [62] for missing values (< 10%) and minor allele frequency (MAF > 0.05), and a total of 11,763 SNPs were considered for GWAS (Additional file 1). SNPs were named considering the physical position in the common bean genome: chromosome (Pv) and genomic position in bp.

### Genome-wide association study

Due to the low frequency of some metabolites in the SDP, only the one or two metabolites of each aglycone found more frequently were considered for GWAS, giving a total of 5 anthocyanins and 5 flavonols (Table 1).

Two different methods for GWAS were considered: single-locus GWAS based on a mixed linear model (MLM; [63]) and multi-locus GWAS based on FASTmrEMMA [64] model. MLM was conducted in Tassel v5.2 [62] and FASTmrEMMA was conducted in the R project using the mrMLM package [57, 65]. For both models, Principal Component Analysis (PCA) and Kinship matrix, obtained by the centered-IBS method, were considered to account for multiple levels of relatedness within the lines included in the panel. QQ plots for the MLM and FASTmrEMMA models were obtained using the rMVP package [58] in R and compared to choose the model that better fits the data.

After Bonferroni correction (α = significance level of 0.05/number of comparisons), a critical threshold of significance (-log10(*p*)) equal to or greater than 5.4 was considered. To verify the robustness of the significant associations identified in the GWAS, the allelic means for each SNP were compared with a T-student test for quantitative traits. For qualitative traits, allelic classes were tested with a chi-squared test. Only significant associations (*p* < 0.05) were considered as a QTN.

### Candidate genes

For candidate genes search, QTNs were analyzed as QTL considering a 350‐Kb window centered for each SNP. In some cases, this window overlapped between adjacent QTL, and they were equally named. QTLs were named using the type of trait (prefix C for the color parameters; prefix Phe for phenolic metabolites), chromosome number, and the start physical position in Mbp. Genes located in each QTL and their associated function were obtained from *P. vulgaris* genome sequence version 2.1 [60, 61]. Among them, the prioritization of candidate genes was based on:

i) search by keywords for molecules involved in the phenolic biosynthetic pathway of common bean based on the annotated gene function using two sources: Phytozome [61], and Kyoto Encyclopedia of Genes and Genomes [66].
ii) genes previously described to be involved in the phenylpropanoid biosynthetic pathway of common bean [11, 12, 26, 31].
iii) the homology of the annotated genes was compared with genes previously described in the phenylpropanoid pathway of *Arabidopsis thaliana* using BLASTp. A total of 50 anthocyanin genes were considered (Additional file 4), 31 of them are structural genes encoding anthocyanin biosynthetic enzymes, 16 are regulatory genes encoding transcriptional factors, and 3 are transport genes necessary for anthocyanin transport from the cytosol to the vacuole [35, 36, 67, 68]. Significant blast scores were considered at an E-value cutoff of ≤ 1E-25 and a percentage of identity > 25%.

## Supplementary Information

**Additional file 1.** Sequential graphic showing the observation index (x-axis) in the lines of the Spanish Diversity Panel for the traits Cya_G, Del_G, Pel_G, Pet_G, Mal_G, Kae_G, Kae_GAcII, Myr_G, Que_G, Que_GAc, L*, b*, and a*.

**Additional file 2.** QQ-plots obtained with MLM and FASTmrEMMA methods for the quantitative traits Cya_G, Del_G, Pel_G, Pet_G, Mal_G, Kae_G, Kae_GAcII, Myr_G, Que_G, Que_GAc, L*, b*, and a*.

**Additional file 3.** QQ-plots obtained with MLM and FASTmrEMMA methods for the qualitative seed color traits White_NB, Cream_NC, Black_NB, Brown_NB, Red_NR, Yellow_NY, and Pattern_NP.

**Additional file 4.** *Arabidopsis thaliana* genes involved in the phenylpropanoid pathway (http://www.arabidosis.net).

**Additional file 5.** Significant alignments (E-value cutoff ≤ 1E-25) derived from the Blastp conducted between the genes located in each QTL and the 50 *Arabidopsis thaliana* genes considered (Additional file 5).

## Data Availability

Data has been publicly deposited. Genotypic characterization analyzed during the current study is available at the European Nucleotide Archive under project ID PRJEB59764 (https://www.ebi.ac.uk/ena/browser/text-search?query=PRJEB59764. Characterization of the seed coat extractable phenolic profile and color analysed during the current study is available at https://doi.org/10.5281/zenodo.7006887.

## Abbreviations

Cya_diG: Cyanindin 3-*O*-diglucoside
Cya_G: Cyanindin 3-*O*-glucoside
Cya_GMal: Cyanindin 3-*O*-6-malonylglucoside
Del_diG: Delphinidin 3,5-*O*-diglucoside
Del_G: Delphinidin 3-*O*-glucoside
F3`H: Flavonol 3’ hydroxylase
F3’,5’H: Flavonol 3’, 5’ hydroxylase
GWAS: Genome wide associations study
Kae: Kaempferol
Kae_G: Kaempferol 3-*O*-glucoside
Kae_GAcI: Kaempherol 3-*O*-acetilglucoside_I
Kae_GAcII: Kaempherol 3-*O*-acetilglucoside_II
Kae_Rut: Kaempferol 3-rutininoside
Kae_XylG: Kaempferol 3-*O*-xyloglucoside
Mal_diG: Malvidin 3,5-*O*-diglucoside
Mal_G: Malvidin 3-*O*-glucoside
Myr: Myricetin
Myr_G: Myricetin 3-*O*-glucoside
Myr_GMal: Myricetin 3-*O*-malonylglucoside
Myr_XylG: Myricetin 3-*O*-xyloglucoside
Pel_diG: Pelargonidin 3,5-*O*-diglucoside
Pel_G: Pelargonidin 3-*O*-glucoside
Pel_GMal: Pelargonidin 3-*O*-6 malonylglucoside
Pel_PenHex: Pelargonidin-*O*-pentosidehexoside
Pet_diG: Petunidin 3,5-*O*-diglucoside
Pet_G: Petunidin 3-*O*-glucoside
QTL: Quantitative trait locus/i
QTN: Quantitative trait nucleotide
Que: Quercetin
Que_G: Quercetin 3-*O*-glucoside
Que_GAc: Quercetin 3-*O*-acetylglucoside
Que_Rut: Quercetin 3-*O*-rutininoside
Que_XylG: Quercetin 3-*O*-xyloglucoside
SDP: Spanish Diversity Panel
