# Supplementary material for: Genome-wide association study for the extractable phenolic profile and coat color of common bean seeds (Phaseolus vulgaris L.)
Source: BMC Plant Biol. 2023 Mar 23;23:158. doi: 10.1186/s12870-023-04177-z (PMC10035135; doi:10.1186/s12870-023-04177-z)
Supplement: Supplementary file 1 — Additional file 1. Sequential graphic showing the observation index (x-axis) in the lines of the Spanish Diversity Panel for the traits Cya_G, Del_G, Pel_G, Pet_G, Mal_G, Kae_G, Kae_GAcII, Myr_G, Que_G, Que_GAc, L*, b*, and a*. [file 12870_2023_4177_MOESM1_ESM.pdf]

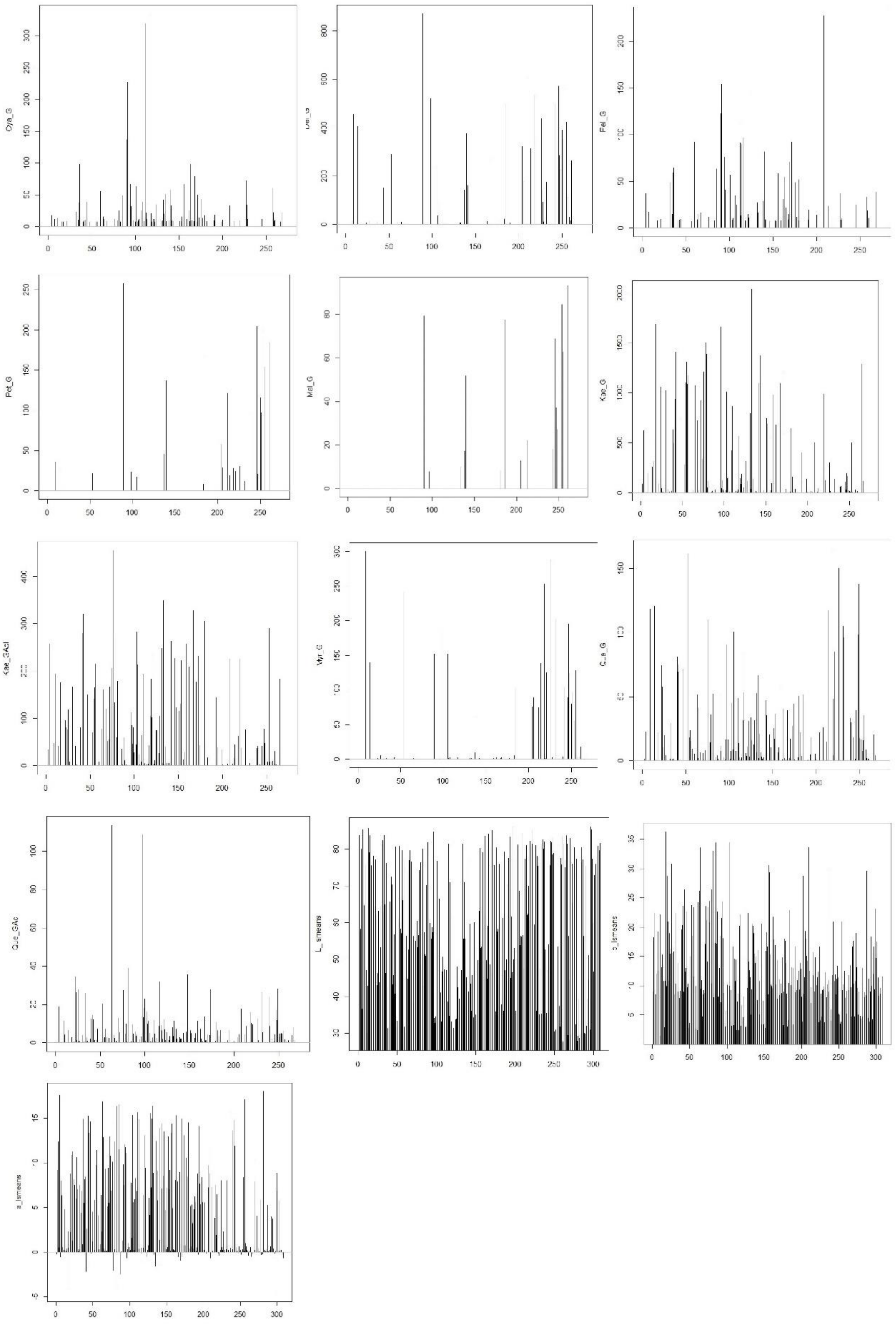

Additional File 1. Sequential graphic showing the observation index (x-axis) in the lines of the Spanish Diversity Panel for the traits Cya_G, Del_G, Pel_G, Pet_G, Mal_G, Kae_G, Kae_GAcII, Myr_G, Que_G, Que_GAc, L*, b*, and a*.
