# Supplementary material for: Genome-wide association study for the extractable phenolic profile and coat color of common bean seeds (Phaseolus vulgaris L.)
Source: BMC Plant Biol. 2023 Mar 23;23:158. doi: 10.1186/s12870-023-04177-z (PMC10035135; doi:10.1186/s12870-023-04177-z)
Supplement: Supplementary file 3 — Additional file 3. QQ-plots obtained with MLM and FASTmrEMMA methods for the qualitative seed color traits White_NB, Cream_NC, Black_NB, Brown_NB, Red_NR, Yellow_NY, and Pattern_NP. [file 12870_2023_4177_MOESM3_ESM.pdf]

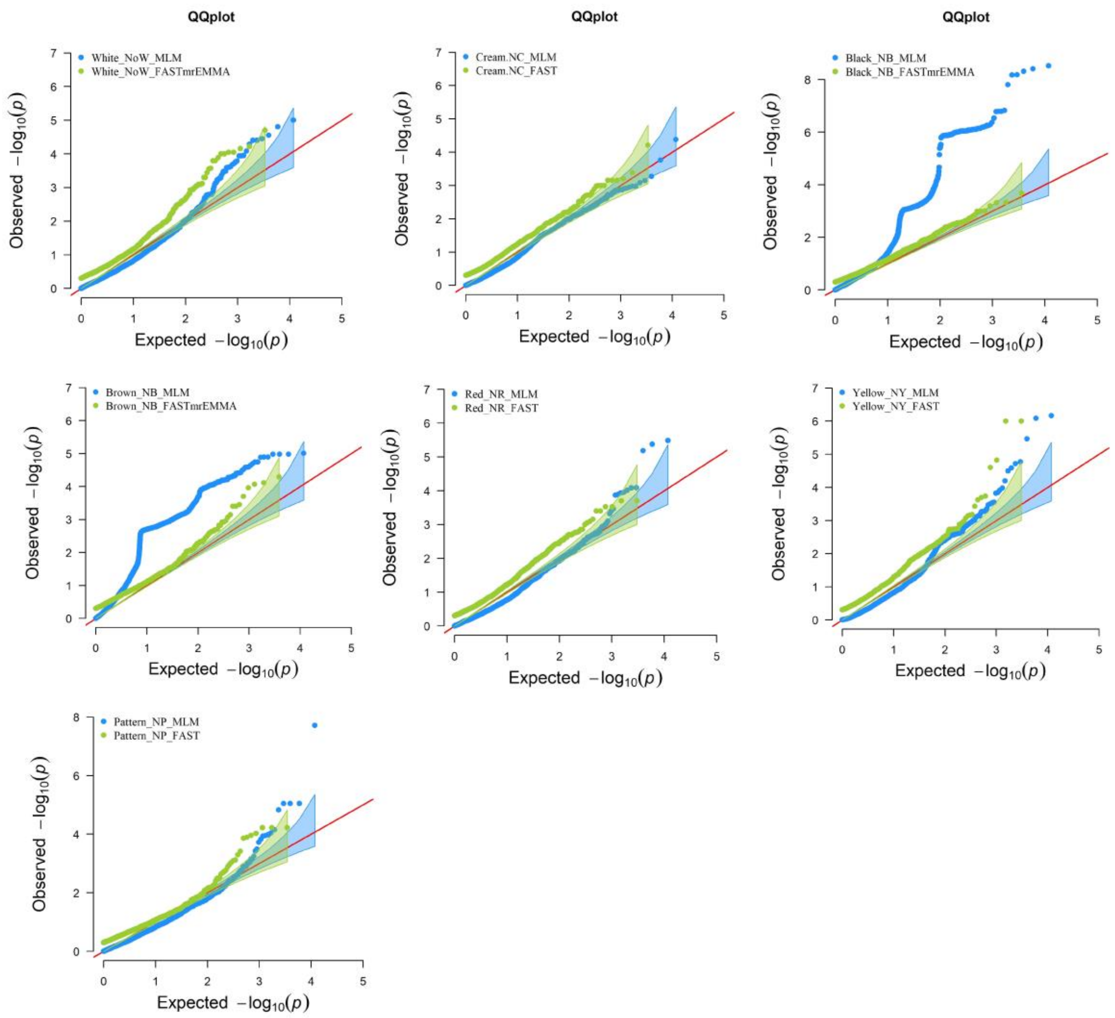

Additional File 3. QQ-plots obtained with MLM and FASTmrEMMA methods for the qualitative seed color traits White_NB, Cream_NC, Black_NB, Brown_NB, Red_NR, Yellow_NY, and Pattern_NP.
